# Supplementary figures and images for: Degree day models to forecast the seasonal phenology of Drosophila suzukii in tart cherry orchards in the Midwest U.S
Source: PLoS One. 2020 Apr 24;15(4):e0227726. doi: 10.1371/journal.pone.0227726 (PMC7182266; doi:10.1371/journal.pone.0227726)

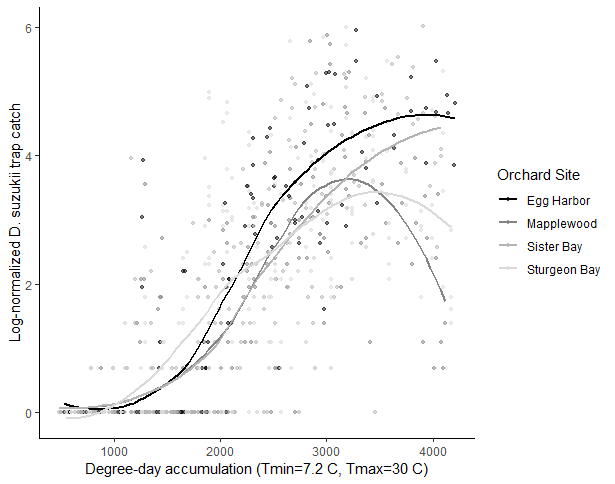

Supplement: S1 Fig — Relationship between log-normalized adult D. suzukii total trap catch for each orchard site/week and weekly cumulative degree days over the four year trapping period. A smoothed fit line shows approximate phenology of D. suzukii for each orchard in this dataset. (TIFF) [file pone.0227726.s001.tiff]
