# Supplementary material for: Degree day models to forecast the seasonal phenology of Drosophila suzukii in tart cherry orchards in the Midwest U.S
Source: PLoS One. 2020 Apr 24;15(4):e0227726. doi: 10.1371/journal.pone.0227726 (PMC7182266; doi:10.1371/journal.pone.0227726)
Supplement: Attachment — Submitted filename: PONE-D-19-35638_markup.pdf [file pone.0227726.s006.pdf]

PLOS ONE

Degree day models to forecast the seasonal phenology of Drosophila suzukii (Diptera: Drosophilidae) in Midwest climate

--Manuscript Draft--

**Manuscript Number:**	PONE-D-19-35638
**Article Type:**	Research Article
**Full Title:**	Degree day models to forecast the seasonal phenology of Drosophila suzukii (Diptera: Drosophilidae) in Midwest climate
**Short Title:**	Phenology of Drosophila suzukii in the Midwest
**Corresponding Author:**	Christelle Guédot<br>University of Wisconsin Madison<br>Madison, Wisconsin UNITED STATES
**Keywords:**	spotted-wing drosophila; phenology; invasive species; population dynamics; monitoring
**Abstract:**	Spotted-wing drosophila, Drosophila suzukii (Matsumura) (Diptera: Drosophilidae), is an invasive economic pest of soft-skinned and stone fruit across the globe. Our study establishes both a predictive generalized linear mixed model (GLMM), and a generalized additive mixed model (GAMM) of the dynamic seasonal phenology of D. suzukii based on four years of adult monitoring trap data in Wisconsin tart cherry orchards collected throughout the growing season. The models incorporate year, field site, relative humidity, and degree days (DD); and relate these factors to trap catch. The GLMM estimated a coefficient of 2.21 for DD/1000, meaning for every increment of 1000 DD, trap catch increases by roughly 9 flies. The GAMM generated a curve based on a cubic regression smoothing function of DD which approximates critical DD points of first adult D. suzukii detection at 1276 DD, above average field populations at 2019 DD, and peak activity at 3180 DD. By incorporating four years of comprehensive seasonal phenology data from the same locations, we introduce robust models capable of using DD to forecast changing adult D. suzukii populations in the field leading to the application of more timely and effective management strategies.
**Order of Authors:**	Matthew T. Kamiyama
	Benjamin Z. Bradford
	Russell L. Groves
	Christelle Guédot
**Additional Information:**	
**Question**	**Response**
**Financial Disclosure**<br><br>Enter a financial disclosure statement that describes the sources of funding for the work included in this submission. Review the submission guidelines for detailed requirements. View published research articles from *PLOS ONE* for specific examples.<br><br>This statement is required for submission and **will appear in the published article** if the submission is accepted. Please make sure it is accurate.	CG received funding for the project from the Wisconsin Department of Agriculture, Trade, and Consumer Protection Specialty Crop Block Grant #16-01. https://datcp.wi.gov/Pages/Homepage.aspx<br>The funders had no role in study design, data collection and analysis, decision to publish, or preparation of the manuscript.

Powered by Editorial Manager® and ProduXion Manager® from Aries Systems Corporation

**Unfunded studies**<br>Enter: *The author(s) received no specific funding for this work.*<br><br>**Funded studies**<br>Enter a statement with the following details:<br>• Initials of the authors who received each award<br>• Grant numbers awarded to each author<br>• The full name of each funder<br>• URL of each funder website<br>• Did the sponsors or funders play any role in the study design, data collection and analysis, decision to publish, or preparation of the manuscript?<br>• **NO** - Include this sentence at the end of your statement: *The funders had no role in study design, data collection and analysis, decision to publish, or preparation of the manuscript.*<br>• **YES** - Specify the role(s) played.<br><br>* typeset	
**Competing Interests**<br><br>Use the instructions below to enter a competing interest statement for this submission. On behalf of all authors, disclose any competing interests that could be perceived to bias this work—acknowledging all financial support and any other relevant financial or non-financial competing interests.<br><br>This statement **will appear in the published article** if the submission is accepted. Please make sure it is accurate. View published research articles from *PLOS ONE* for specific examples.	The authors have declared that no competing interests exist.

**NO authors have competing interests**<br><br>Enter: *The authors have declared that no competing interests exist.*<br><br>**Authors with competing interests**<br><br>Enter competing interest details beginning with this statement:<br><br>*I have read the journal's policy and the authors of this manuscript have the following competing interests: [insert competing interests here]*<br><br>* typeset	
**Ethics Statement**<br><br>Enter an ethics statement for this submission. This statement is required if the study involved:<br><br>• Human participants<br>• Human specimens or tissue<br>• Vertebrate animals or cephalopods<br>• Vertebrate embryos or tissues<br>• Field research<br><br>Write "N/A" if the submission does not require an ethics statement.<br><br>General guidance is provided below. Consult the submission guidelines for detailed instructions. **Make sure that all information entered here is included in the Methods section of the manuscript.**	N/A

**Format for specific study types**<br><br>**Human Subject Research (involving human participants and/or tissue)**<br>• Give the name of the institutional review board or ethics committee that approved the study<br>• Include the approval number and/or a statement indicating approval of this research<br>• Indicate the form of consent obtained (written/oral) or the reason that consent was not obtained (e.g. the data were analyzed anonymously)<br><br>**Animal Research (involving vertebrate animals, embryos or tissues)**<br>• Provide the name of the Institutional Animal Care and Use Committee (IACUC) or other relevant ethics board that reviewed the study protocol, and indicate whether they approved this research or granted a formal waiver of ethical approval<br>• Include an approval number if one was obtained<br>• If the study involved *non-human primates*, add *additional details* about animal welfare and steps taken to ameliorate suffering<br>• If anesthesia, euthanasia, or any kind of animal sacrifice is part of the study, include briefly which substances and/or methods were applied<br><br>**Field Research**<br><br>Include the following details if this study involves the collection of plant, animal, or other materials from a natural setting:<br>• Field permit number<br>• Name of the institution or relevant body that granted permission	
**Data Availability**<br><br>Authors are required to make all data underlying the findings described fully available, without restriction, and from the time of publication. PLOS allows rare exceptions to address legal and ethical concerns. See the PLOS Data Policy and FAQ for detailed information.	Yes - all data are fully available without restriction

Powered by Editorial Manager® and ProduXion Manager® from Aries Systems Corporation

A Data Availability Statement describing where the data can be found is required at submission. Your answers to this question constitute the Data Availability Statement and **will be published in the article**, if accepted.<br><br>**Important:** Stating 'data available on request from the author' is not sufficient. If your data are only available upon request, select 'No' for the first question and explain your exceptional situation in the text box.<br><br>Do the authors confirm that all data underlying the findings described in their manuscript are fully available without restriction?	
**Describe where the data may be found in full sentences. If you are copying our sample text, replace any instances of XXX with the appropriate details.**<br><br>• If the data are **held or will be held in a public repository**, include URLs, accession numbers or DOIs. If this information will only be available after acceptance, indicate this by ticking the box below. For example: *All XXX files are available from the XXX database (accession number(s) XXX, XXX.*).<br>• If the data are all contained **within the manuscript and/or Supporting Information files**, enter the following: *All relevant data are within the manuscript and its Supporting Information files.*<br>• If neither of these applies but you are able to provide **details of access elsewhere**, with or without limitations, please do so. For example:<br><br>*Data cannot be shared publicly because of [XXX]. Data are available from the XXX Institutional Data Access / Ethics Committee (contact via XXX) for researchers who meet the criteria for access to confidential data.*<br><br>*The data underlying the results presented in the study are available from (include the name of the third party*	All relevant data are within the manuscript and its Supporting Information files.

and contact information or URL).<br>• This text is appropriate if the data are owned by a third party and authors do not have permission to share the data.<br><br>* typeset	
Additional data availability information:	Tick here if the URLs/accession numbers/DOIs will be available only after acceptance of the manuscript for publication so that we can ensure their inclusion before publication.

Powered by Editorial Manager® and ProduXion Manager® from Aries Systems Corporation

Title Page Click here to access/download;Manuscript;PLOS ONE Title Page.docx

Degree day models to forecast the seasonal phenology of *Drosophila suzukii* (Diptera: Drosophilidae) in Midwest climate

Matthew T. Kamiyama[1,2], Benjamin Z. Bradford[2], Russell L. Groves[2], and Christelle Guédot[2*]

[1] Research Institute for Sustainable Humanosphere, Division of Applied Biosciences, Kyoto University, Kyoto, Japan

[2] Department of Entomology, University of Wisconsin-Madison, Madison, Wisconsin, United States of America

*Corresponding author

Email: guedot@wisc.edu

Manuscript
Click here to access/download;Manuscript;PLOS ONE Manuscript Body.docx

Abstract

Spotted-wing drosophila, *Drosophila suzukii* (Matsumura) (Diptera: Drosophilidae), is an invasive economic pest of soft-skinned and stone fruit across the globe. Our study establishes both a predictive generalized linear mixed model (GLMM), and a generalized additive mixed model (GAMM) of the dynamic seasonal phenology of *D. suzukii* based on four years of adult monitoring trap data in Wisconsin tart cherry orchards collected throughout the growing season. The models incorporate year, field site, relative humidity, and degree days (DD); and relate these factors to trap catch. The GLMM estimated a coefficient of 2.21 for DD/1000, meaning for every increment of 1000 DD, trap catch increases by roughly 9 flies. The GAMM generated a curve based on a cubic regression smoothing function of DD which approximates critical DD points of first adult *D. suzukii* detection at 1276 DD, above average field populations at 2019 DD, and peak activity at 3180 DD. By incorporating four years of comprehensive seasonal phenology data from the same locations, we introduce robust models capable of using DD to forecast changing adult *D. suzukii* populations in the field leading to the application of more timely and effective management strategies.

Introduction

Spotted-wing drosophila, *Drosophila suzukii* (Matsumura) (Diptera: Drosophilidae), is an invasive economic pest of soft-skinned and stone fruit in North America, South America, and Europe [1, 2, 3, 4]. Female *D. suzukii* possess a distinctive, serrated ovipositor that allows them to infest still ripening and ripe fruit [5], unlike other species of drosophilids which can only target overripe, rotting, or damaged fruit. Along with this unique morphological feature, *D.*

suzukii have high rates of reproduction, fast generation times, and quickly adapt to variable climates making them a formidable economic pest [2, 6, 7]. Damage from *D. suzukii* to susceptible fruit crops in Western U.S. states can result in annual losses up to $511 million [8]. In Minnesota, crop damage resulting from *D. suzukii* was projected at $2.2 million annually in raspberry alone [9].

Rigorous insecticide application regimens are the most prominent means of *D. suzukii* management in agricultural areas of high pest pressure [10, 11]. In the Midwest, frequent insecticide treatments are implemented in *D. suzukii* affected farms and orchards during the field season with applications surpassing five occasions when necessary [12, 13]. Chemical control is a costly management option not only monetarily, but also environmentally [8, 11, 14, 15]. Growers commonly apply insecticides which specifically target adult *D. suzukii* and rotate insecticides which incorporate active ingredients that have different modes of action, which helps minimize the number of treatments per season and reduces the risk of developed resistance [12, 13]. A better understanding of this pest's seasonal phenology and the related underlying mechanisms is an instrumental step in continuously building more efficient and effective integrated pest management strategies against *D. suzukii*.

Modeling the seasonal population variations of pests from field monitoring data can help determine periods of high pest abundance [16]. Phenology models are particularly effective in describing the population dynamics of pests which maintain relatively few and synchronous generations during the year [17, 18]. *Drosophila suzukii,* however, is a highly prolific pest which has rapid rates of reproduction and several overlapping generations [7, 19]. Previously developed *D. suzukii* predictive phenology models navigated this caveat by generating developmental stage-based models [20], or physiological age-structured models approximated by degree-days (DD)

[21]. Abiotic factors such as temperature (DD) and relative humidity (RH) are major drivers of *D. suzukii* development activity [7, 22, 23, 24], and incorporating these effects allows for more descriptive models. Population modeling of *D. suzukii* can provide a better understanding of this pest's population trends in a regionally specific sense, having potential management implications such as timing insecticide treatments with predictions of high pest pressure [20, 21]. Models have previously been produced from data obtained in the Pacific Coast, North Carolina, and Michigan regions [13, 20], but they do not accurately describe *D. suzukii* population dynamics observed in ~~the~~ Wisconsin. No model incorporating DD or RH describing the pest's seasonal population variation in the Midwest is currently available.

Our study establishes both a predictive generalized linear mixed model, and a generalized additive mixed model of the dynamic seasonal phenology of *D. suzukii* based on four years of adult monitoring trap data collected in Wisconsin tart cherry orchards during the growing season in relation to DD and RH. By incorporating four years of comprehensive seasonal phenology data from the same locations, we present two models using DD and RH which forecast changing adult *D. suzukii* populations in the field in Wisconsin.

Materials and methods

Adult seasonal phenology

Field sites

To assess the seasonal phenology of *D. suzukii*, we sampled four tart cherry orchards from 2015-2018 in northeastern Wisconsin, U.S., located near the urban centers of Maplewood, Sturgeon Bay, Egg Harbor, and Sister Bay. Each year, a total of 13 traps were placed across

these locations, and each trap location was re-sampled each year. Three traps were placed in Maplewood, five traps in Sturgeon Bay, two traps in Egg Harbor, and three traps in Sister Bay. All of the orchards grew the 'Montmorency' cultivar, while one location grew 'Montmorency' and 'Balaton'. All orchards were conventionally managed for *D. suzukii* by rotating pyrethroid and organophosphate insecticides, averaging four to five applications throughout the growing season. Adult *D. suzukii* field monitoring from 2015 – 2018 typically began in mid-May, and continued until late-August, two weeks post tart cherry harvest.

Monitoring traps

Scentry traps baited with Scentry *D. suzukii* attractant lures (Scentry Biologicals Inc., Billings, MT, U.S.) were used to monitor adult populations of *D. suzukii*. At the bottom of each trap, a drowning solution containing 200 mL of water, 0.8 g of boric acid, and 2 – 3 mL of unscented dish soap (Seventh Generation Inc., Burlington, VT, U.S.) was added to kill and preserve collected specimens. Monitoring traps were placed in the lower canopy fruiting zone of tart cherry trees. Every week, the trap contents were emptied and deposited in 70% ethanol, then returned to the laboratory where adult *D. suzukii* from each trap were counted. The drowning solution was replaced weekly, and the Scentry lures were substituted every four weeks based on the manufacturer's recommendations.

Temperature and humidity

Temperature data for each site from 2015 – 2018 were retrieved from the PRISM Climate Group [25] by entering the GPS coordinates of each site. Minimum, mean, and maximum temperatures were retrieved and degree-days (DD) were calculated using a lower threshold of

7.2° C and upper threshold of 30° C, as *D. suzukii* development ceases beyond these temperature boundaries [7]. Cumulative weekly DD totals were computed for each site from January 1st of each year until the end of *D. suzukii* monitoring. Relative humidity (RH) data for each site from 2014 – 2018 were retrieved from the Michigan State Enviroweather website [26]. Data were downloaded from four weather stations located 1-7 km from the monitoring sites. Relative humidity was averaged for each week during the monitoring periods.

Statistical analysis

Generalized linear mixed model

A generalized linear mixed model (GLMM) was generated from *D. suzukii* adult trap catch, DD, RH, year, and site data. The model is based on negative binomial regression fit by maximum likelihood using the *glmer* function in the *lme4* package of R version 3.5.3 [27]. Year, site, and the year*site interaction were incorporated into the model as random effects, and DD and RH were added as fixed effects. Degree days were divided by 1000 to fit an appropriate scale with the other effects. The GLMM can be described as follows:

$$Y_{ij} \sim \text{NegBin}\,[\mu_{ij(abc)}]$$

$$g[\mu_{ij(abc)}] = log\,[\mu_{ij(abc)}] = \mu_{ij} + \beta_i + \beta_j + \varepsilon_a + \varepsilon_b + \varepsilon_c + \delta_{ij}$$

$$\varepsilon_a \sim N\,(0, \sigma^2{}_a),$$

$$\varepsilon_b \sim N\,(0, \sigma^2{}_b),$$

$$\varepsilon_c \sim N\,(0, \sigma^2{}_c),$$

$$\delta_{ij} \sim N\,(0, \sigma^2)$$

In this representation of the model, (Y_{ij}) is the estimate of weekly adult *D. suzukii* trap catch as a function of non-linear time (DD) (x_i) and RH (x_j) with year (ε_a), site (ε_b), and year*site (ε_c) added

as random effects. Coefficients β_i and β_i correspond to the trap catch explained by DD and RH respectively, and δ_{ij} represents the residual error of the trap catch estimation. The GLMM allowed for random effects to be accounted for prior to estimating the regression coefficients. While separating random from mixed effects creates a much more interpretable model, this approach limits its flexibility as the coefficient estimate will be solely increasing or decreasing (Wood, 2006). The GLMM as fit will only predict an increasing or decreasing trap catch throughout the entire seasonal phenology of *D. suzukii* based on changing DD, but can predict a specific trap catch given a unique DD estimate.

Model diagnostics

Scatter plots were produced for trap catch at each site from 2015 – 2018. These dot plots mirrored quantile-quantile plots of the same data, meaning the effect of site and year could be assumed to be random and of equal variance [28]. The full GLM model (including RH as a fixed effect with all random effects) and the reduced model (removing RH and the interaction between random effects) were compared to determine which model best fit the data. An Akaike information criterion (AIC) and a likelihood ratio test (LRT) were run to determine the best fit model between the two models. The inclusion of all parameters was justified as the full model had slightly more explanatory power when comparing the two models (full model AIC = 4875.7, reduced model AIC = 4883.7, LRT $p = 0.002$). The full GLM model was also selected because we recognize RH as an important contributing abiotic factor in *D. suzukii* population dynamics [22, 23, 24].

Generalized additive mixed model

A generalized additive mixed model (GAMM) was also generated from the same *D. suzukii* adult trap catch, DD, RH, year, and site data. The model is based on negative Poisson regression with a log-link using the *predict.gam* function in the *lme4* and *mgcv* packages of R version 3.5.3. Degree days, year, site, and the year*site interaction were incorporated into the model as random effects, and RH was added as a fixed effect. The GAMM can be described as follows:

$$Y_{ij} \sim \text{Poisson}\ [\mu_{ij(abc)}]$$

$$g[\mu_{ij(abc)}] = log\ [\mu_{ij(abc)}] = f\,(d_i) + \beta_j + \varepsilon_{(a)} + \varepsilon_{(b)} + \varepsilon_{(c)} + \delta_{ij}$$

$$\varepsilon_{(a)} \sim \text{N}\ (0, \sigma^2{}_a),$$

$$\varepsilon_{(b)} \sim \text{N}\ (0, \sigma^2{}_b),$$

$$\varepsilon_{(c)} \sim \text{N}\ (0, \sigma^2{}_c),$$

$$\delta_{i\,j} \sim \text{N}\ (0, \sigma^2)$$

In this model, (Y_{ij}) is used to describe the seasonal pattern of adult *D. suzukii* through non-linear time (DD/1000) (x_i) as a random effect and RH (x_j) as a fixed effect. Coefficient β_i corresponds to the trap catch explained by RH and $f\,(d_i)$ is included in the model as a penalized cubic regression smoothing function of DD. Year (ε_a), site (ε_b), and year*site (ε_c) were also added as random effects, and δ_{ij} represents the residual error of the trap catch estimation. Using the GAMM, ~~were~~ have the ability to estimate underlying trends of the *D. suzukii* population throughout the growing season. Overall, the GAMM has less interpretability than the GLMM (Wood, 2006), but has more flexibility in attributing DD to critical points in the seasonal phenology of *D. suzukii* including first adult detection, dramatic increase in population size, and peak activity.

Model diagnostics

Scatter plots were produced for trap catch at each site from 2015 – 2018 which mirrored quantile-quantile plots of the same data, and allowed us to assume the effect of site and year to be random and of equal variance [28]. Similar to the GLMM, the GAM full model (including RH as a fixed effect with all random effects) and the reduced model (removing RH and the interaction between random effects) were compared to determine the best fitting model. A generalized cross-validation (GCV) score was generated for both models using the *mgcv* package of R version 3.5.3. The GCV score is used to measure model smoothness selection as well as estimate prediction error. Generalized cross-validation scores are comparable to an AIC value in that a lower score indicates a better fitting model. Our full GAM model had a GSV score of 1.63 and the reduced model had a score of 1.77, so the full model was selected for analysis.

Results

The field populations of *D. suzukii* generally increased with DD during the field season, then peaked at the accumulation of about 3000 DD when looking at the log-transformed trap catch data from each site from 2015 – 2018 (Fig 1). Log-normalized average trap catch for each orchard site from the four year study period is presented with best fit curves to better illustrate the trap catch variance between each orchard (S1 Fig). A simple linear regression explained that the log-normalized trap catch had a slight positive correlation with RH ($t = 5.50$, $p < 0.001$, $R^2 = 0.04$, $y = 2.37x – 146.21$) (Fig 2).

Fig 1. Weekly adult *D. suzukii* trap catch over degree days. Relationship between log-normalized adult *D. suzukii* total trap catch for each trap/week and weekly cumulative degree

days over the four year trapping period. A smoothed fit line shows approximate phenology of *D. suzukii* for this dataset.

Fig 2. Weekly adult *D. suzukii* trap catch over relative humidity. Relationship between log-normalized total adult *D. suzukii* trap catch for each trap/week and mean weekly relative humidity at the trap location over the four year trapping period. A positive relationship between trap catch and increasing relative humidity is shown.

The GLMM analyzed trap catch from each site over the four year period. The random effect of year explained 24% of the total variance in *D. suzukii* trap catch, while the effect of site accounted for 5%, and the year * site interaction was responsible for 18% of the trap catch variability of the random effects. The coefficient estimates are 2.21 ($Z = 23.95$, $p < 0.001$) for DD/1000 and 0.03 ($Z = 0.02$, $p = 0.08$) for RH. This model estimates an increase of $e^{(2.21)}$ or about nine flies per every 1000 DD accumulated and an increase of $e^{(0.03)}$ or about one fly per every increase of one percent RH.

A smoothed curve plot (Fig 3) was generated by the GAMM, incorporating trap catch data from all sites over the four years to analyze seasonal population trends of *D. suzukii*. The plot suggests first adult detection occurs at 1276 DD and periods of above average trap catch began at 2019 DD and continued until 4707 DD. A very distinguishable interval of *D. suzukii* population increase occurs between 1549 DD and 3180 DD, which roughly corresponds with early July through early September in Wisconsin.

Fig 3. GAMM predicted *D. suzukii* population dynamics over degree days. GAMM generated smooth curve plot of adult *D. suzukii* trap catch total for each site in relation to degree day accumulation (DD) over the four year trapping period. Critical DD values are labelled and boxed on the curve. Conditional modes (CM) measure the population level estimations given the effects (positive CM = higher than average, negative CM = lower than average).

The GAMM produced critical DD values (1276, 1549, 2019, and 3180) were plugged into the GLMM with their corresponding RH values and a predicted trap catch was generated (Table 1). Relative humidity values were taken from the sampling event closest in DD to the critical DD values from each site and year and were averaged. The true *D. suzukii* trap catches were averaged from each site and year from the sampling event closest to each critical DD value (Table 1). One sample *t*-tests were then performed to determine the validity of the GLMM by comparing the predicted mean trap catches to the true mean trap catches. The GLMM predicted trap catches at 1276 and 3180 DD were statistically similar to the corresponding true trap catches at $p < 0.05$, but the predicted trap catches at 1549 and 2019 DD were significantly different than the true trap catches at those respective DD at $p < 0.05$.

Table 1. Comparisons of GLMM predicted adult *D. suzukii* trap catch with true trap catch.

DD	RH	GLMM trap catch	True trap catch	95% CI	*t*	*df*	*p*
1276	70.7	1.2*	3.1 ± 1.2	0.7 – 5.6	1.6	51	0.1
1549	71.9	2.5	1.2 ± 0.4	0.4 – 1.9	-3.7	51	0.001
2019	71.7	6.6	13.7 ± 3.5	6.6 – 20.7	2.0	51	0.05
3180	75.4	87.1*	95.9 ± 16.2	63.2 – 128.6	0.5	41	0.6

Drosophila suzukii mean trap catch estimation resulting from the GLMM (generalized linear mixed model) compared to the actual mean trap catch (± SE) from all sites from 2014 – 2018 at

each of the critical DD (degree day) value, and corresponding average RH (relative humidity). Results from the one sample *t*-test are also included: *t*-test statistic (*t*), degrees of freedom (*df*), *p*-value (*p*), and 95% confidence interval (CI) of the true mean trap catch.

* indicates a GLMM predicted *D. suzukii* trap catch statistically similar to the true trap catch at the corresponding DD and RH (one sample *t*-test: $p < 0.05$).

Discussion

The models presented from this research suggest that seasonal *D. suzukii* populations in Wisconsin tart cherry orchards follow predictable patterns based on DD and RH. Our models integrate a robust dataset comprising four years of *D. suzukii* trap catches from the same tart cherry orchards in eastern Wisconsin. The GLMM model is able to forecast adult *D. suzukii* numbers as weekly trap catches, which is useful when needing quantitative estimates of field populations. The GAMM model generated a smoothed curve plot representing *D. suzukii* population tendencies throughout the growing season, which is valuable in determining periods of high or low risk for elevated *D. suzukii* numbers and phases of rapid population increase or decrease. The field population trends described from this work are similar to those in other studies in Wisconsin fruit crops which report *D. suzukii* first detection in the early summer, peak in activity in the late summer, and decreasing populations in the early fall [23, 29]. These seasonal population trends differ from previous *D. suzukii* phenology work done in California which describe a bimodal population distribution with peak numbers occurring in the early summer and late fall, and a decrease in population during the mid to late summer [30]. It is possible this mid-season period of quiescence in California *D. suzukii* populations is explained

by the flies' exposure to high temperatures and low humidity; similar conditions are suggested in inducing a vernal-estival dormancy in other Diptera, such as the olive fruit fly (*Bactrocera oleae*) [31].

The findings of our research are comparable to *D. suzukii* population modeling studies from Michigan, Washington, Oregon, and North Carolina [13, 20]. In the work done by Wiman et al., 2014, estimates for California and North Carolina populations of *D. suzukii* followed a bimodal trend of early summer increase, mid-summer decrease, then late summer increase, whereas the Oregon *D. suzukii* estimates were more analogous to our results of a single peak of high activity late in the field season. However, the data used to predict *D. suzukii* populations in Oregon were largely different than our Wisconsin data; the highest *D. suzukii* trap catch was 200 adults per week in Wisconsin compared to 27 in Oregon, and around 4000 DD were accumulated in Wisconsin during the field season while roughly 1900 DD (Tmin = 4° C, no Tmax) were accumulated in Oregon [20]. A predictive generalized additive model (GAM) generated from seven years of *D. suzukii* trap catch data in Michigan blueberry forecasted a similar population trend to our findings of first *D. suzukii* detection occurring in the early/mid-summer, populations peaking in the late summer, then numbers decreasing in the late summer/early fall [13]. The GAM developed by Leach et al., 2019 was based off of calendar day, as opposed to our GAMM based off DD, and incorporated parameters including: first D. suzukii capture, spring activity, prior year max activity, the number of days below 0°C and above 10°C in spring and winter, the number of days above 60% RH, and the number of days above 28°C. The predictive models presented from our research are supported by regionally specific data obtained from Wisconsin, and are the first which can forecast field *D. suzukii* levels in the Midwestern US relative to DD and RH.

Our DD based models allow for a more precise estimation of field *D. suzukii* populations than calendar day models alone, but incorporating DD may restrict the ability to discern between the different late season generations of this multivoltine pest [7, 20] One advantage of structuring models with DD is that they do not assume consistent temperatures from year to year on the same date, and can be more easily applied to other locations with dissimilar climates. Since the life cycle of *D. suzukii* is so heavily dependent on temperature [7, 23, 32], we assumed there would be less variability in trap catch from year to year for a given DD as opposed to calendar day. Relative humidity data from each site each year were also incorporated into the models as RH also plays an important role in *D. suzukii's* seasonal activity [22, 23, 24]. Several additional factors such as photoperiod, host crop availability, and presence of natural enemies may impact the phenology of *D. suzukii* as well [21, 33, 34]. Future models directed towards approximating the seasonal phenology of *D. suzukii* should consider all significant biotic and abiotic influences, including factors that remain unresolved such as *D. suzukii* overwintering habits and native biological control.

This research adds a valuable new tool to crop protection against *D. suzukii* by predicting their field population trends in relation to DD an RH, allowing for the preemptive implementation of integrated pest management strategies, or improved trap deployment. Our models best apply to growers in the Midwest who farm crops susceptible to *D. suzukii* in locations with a known presence of the pest, or areas of potential invasion. Currently, growers in the Midwest are advised to begin management practices at first detection of *D. suzukii* in their region [35], but in some cases, control measures in some fruit crops may be delayed until fruits reach a susceptible stage of development [36]. The GLMM is slightly more accurate in estimating *D. suzukii* populations in the early and later portions of the season (Table 1), meaning

first detection and peak activity can be more precisely predicted than mid-season numbers in affected Midwest fruit crop farms. According to the GAMM, the first detection of *D. suzukii* is estimated to occur at 1262 DD, meaning control measures should be initiated when that DD accumulation is reached in a given year. For reference, 1276 DD fell most closely on June 26th, 24th, 23rd, and 22nd in 2015, 2016, 2017, and 2018 respectively. There also exists a specific period in the mid-summer (1549 DD) in which *D. suzukii* populations begin to increase, then finally peak in the early fall (3180 DD). Extensive management strategies implemented during the brief 1276-1549 DD period when *D. suzukii* levels are still low may help to alleviate the impending rapid increase of *D. suzukii* in the field forecasted by our models. Wiman et al., 2016 also alludes to the importance of targeting the initial *D. suzukii* adults for control to reduce the opportunity for high populations to build up and fruit damage to increase throughout the season. Though, any management practice directed to a given crop at this time should be imposed only when susceptible fruit is present on the crop, otherwise the attempt may be meaningless. We understand that these models are regionally limited, derived from the specific, and varying factors pertaining to Wisconsin tart cherry orchards. However, continued work on population modeling of *D. suzukii* in different climatic areas is an integral step towards the effective and efficient management of this pest in all invaded regions.

Acknowledgments

We thank all participating growers for access to their orchards to set up *D. suzukii* monitoring traps. We also thank Matt Stasiak from the University of Wisconsin Door County Peninsular Agricultural Research Station for helping with trapping efforts and providing us with archived trap catch data. We give special thanks to Zach Schreiner, Janet van Zoeren, and

Benjamin Jaffe for their help with data processing and field work. This project was funded by the Wisconsin Department of Agriculture, Trade, and Consumer Protection Specialty Crop Block Grant #16-01.

References

1. Bolda MP, Goodhue RE, Gray ME. Spotted wing drosophila: potential economic impact of a newly established pest. Agricultural and Resource Economics Update, University of California, Giannini Foundation. 2016;13: 5-8.

2. Walsh DB, Bolda MP, Goodhue RE, Dreves AJ, Lee J, Bruck DJ, Walton VM, Sally DO, Zalom FG. *Drosophila suzukii* (Diptera: Drosophilidae): Invasive pest of ripening soft fruit expanding its geographic range and damage potential. J Integr Pest Manag. 2011;2: 1-7.

3. Calabria G, Maca J, Bachli G, Serra L, Pascual M. First records of the potential pest species *Drosophila suzukii* (Diptera: Drosophilidae) in Europe. J Appl Ecol. 2012;136: 139-147.

4. Depra M, Poppe JL, Schmitz HJ, De Toni DC, Valente VLS. (2014). The first records of the invasive pest *Drosophila suzukii* in the South American continent. J Pest Sci. 2014;87: 379-383.

5. Hauser M. A historic account of the invasion of *Drosophila suzukii* Matsumura (Diptera: Drosophilidae) in the continental United States, with remarks on their identification. Pest Manag Sci. 2011;67: 1352-1357.

6. Beers EH, Van Steenwyk RA, Shearer PW, Coates WW, Grant JA. Developing *Drosophila suzukii* management programs for sweet cherry in the western United States. Pest Manag Sci. 2011;67: 1386-1395.

7. Tochen S, Dalton DT, Wiman N, Hamm C, Shearer PW, Walton VM. Temperature-related development and population parameters for *Drosophila suzukii* (Diptera: Drosophilidae) on cherry and blueberry Environ Entomol. 2014;43: 501-510.

8. Goodhue RE, Bolda M, Farnsworth D, Williams JC, Zalom FG. Spotted wing drosophila infestation of California strawberries and raspberries: economic analysis of potential revenue losses and control costs. Pest Manag Sci. 2011;67: 1396-1402.

9. Digiacomo G, Hadrich J, Hutchison WD, Peterson H, Rogers M. Economic impact of spotted-wing drosophila (Diptera: Drosophilidae) yield loss on Minnesota raspberry farms: A grower survey. J Integr Pest Manag. 2019;10: 11.

10. Van Timmeren, S, Isaacs R. Control of spotted wing drosophila, *Drosophila suzukii*, by specific insecticides and by conventional and organic crop protection programs. Crop Prot. 2013;54: 126-133.

11. Diepenbrock LM, Rosensteel DO, Hardin JA, Sial AA, Burrack HJ. Season-long programs for control of *Drosophila suzukii* in southeastern U.S. blueberries. Crop Prot. 2016;81: 76-84.

12. Kamiyama MT, Schreiner Z, Guédot C. Diversity and abundance of natural enemies of *Drosophila suzukii* in Wisconsin, USA fruit farms. BioControl. 2019;64: 665-676.

13. Leach H, Van Timmeren S, Wetzel W, Isaacs R. Predicting within- and between-year variation in activity of the invasive spotted wing drosophila (Diptera: Drosophilidae) in a temperate region. Environ Entomol. 2019;48: 1223-1233.

14. Bridges CM. Long-term effects of pesticide exposure at various life stages of the southern leopard frog (*Rana sphenocephala*). Arch Environ Contam Toxicol. 2000;39; 91-96.

15. Epstein DL, Zack RS, Brunner JF, Gut L, Brown JJ. Effects of broad-spectrum insecticides on epigeal arthropod biodiversity in Pacific Northwest apple orchards. Environ Entomol. 2000;29: 340-348.

16. Frost KE, Esker PD, Van Haren R, Kotolski L, Groves RL. Factors influencing aster leafhopper (Hemiptera: Cicadellidae) abundance and aster yellows phytoplasma infectivity in Wisconsin carrot fields. Environ Entomol. 2013;42: 477-490.

17. Reissig W, Tette J. Apple maggot (Diptera: Tephritidae) monitoring in an apple pest management program. Can Entomol. 1979;111: 621-625.

18. Teixeira LAF, Polavarapu S. Postdiapause development and prediction of emergence of female blueberry maggot (Diptera: Tephritidae). Environ Entomol. 2001;30: 925-931.

19. Asplen MK, Anfora G, Biondi A, Choi D-S, Chu D, Daane KM, Gibert P, Gutierrez AP, Hoelmer KA, Hutchinson WD, Isaacs R, Jiang Z-L, Kárpáti Z, Kimura MT, Pascual M, Philips CR, Plantamp C, Ponti L, **Vétek** G, Vogt H, Walton VM, Yu Y, Zappalà L, Desneux N. Invasion biology of spotted wing drosophila (*Drosophila suzukii*): a global perspective and future priorities. J Pest Sci. 2015;88: 469-494.

20. Wiman NG, Walton VM, Dalton DT, Anfora G, Burrack HJ, Chiu JC, Daane KM, Grassi A, Miller B, Tochen S, Wang,X, Ioriatti C. Integrating temperature- dependent life table data into a matrix projection model for *Drosophila suzukii* population estimation. PLoS ONE. 2014;9: e106909.

21. Wiman NG, Dalton DT, Anfora G, Biondi A, Chiu JC, Daane KM, Gerdeman B, Gottardello A. Hamby KA, Isaacs R, Grassi A, Ioriatti C, Lee JC, Miller B, Rossi Stacconi MV, Shearer PW. Tanigoshi L, Wang X, Walton VM. *Drosophila suzukii* population response to environment and management strategies. J Pest Sci. 2016;89: 653-665.

22. Tochen S, Woltz JM, Dalton DT, Lee JC, Wiman NG, Walton VM. Humidity affects populations of *Drosophila suzukii* (Diptera: Drosophilidae) in blueberry. J Appl Entomol. 2016;140: 47-57.

23. Guédot C, Avanesyan A, Hietala-Henschell K. Effect of temperature and humidity on the seasonal phenology of *Drosophila suzukii* (Diptera: Drosophilidae) in Wisconsin. Environ Entomol. 2018;47: 1365-1375.

24. Jaffe BD, Guédot C. Vertical and temporal distribution of spotted- wing drosophila (*Drosophila suzukii*) and pollinators within cultivated raspberries. Pest Manag Sci. 2019 Jan 20. doi: 10.1002/ps.5343.

25. PRISM Climate Group. 2019 Northwest Alliance for Computational Science & Engineering, Corvallis, OR. 2019. Available from: http://prism.oregonstate.edu/

26. Enviroweather. Weather-based pest, natural resource, and production management tools. East Lansing, MI. 2019. Available from: https://enviroweather.msu.edu/

27. R Core Team. R: a language and environment for statistical computing. R Foundation for Statistical Computing. Vienna, Austria. 2018. Available from: https://www.r-project.org/

28. Wood SN. Generalized Additive Models: An Introduction with R. Chapman and Hall/CRC; 2006.

29. Pelton E, Gratton C, Guédot C. Susceptibility of cold hardy grapes to *Drosophila suzukii* (Diptera: Drosophilidae). J Appl Entomol. 2017;141: 644-652.

30. Wang X-G, Stewart TJ, Biondi A, Chavez BA, Ingels C, Caprile J, Grant JA, Walton VM, Daane KM. Population dynamics and ecology of *Drosophila suzukii* in Central California. J Pest Sci. 2016;89: 701-712.

31. Daane KM, Johnson MW. Olive fruit fly: Managing an ancient pest in modern times. Annu Rev Entomol. 2010;55: 151-169.

32. Hamby KA, Bellamy DE, Chiu JC, Lee JC, Walton VM, Wiman NG, York RM, Biondi A. Biotic and abiotic factors impacting development, behavior, phenology, and reproductive biology of *Drosophila suzukii*. J Pest Sci. 2016;89: 605-619.

33. Lee, J. C., Dreves, A. J., Cave, A. M., Kawai, S., Isaacs, R., Miller, J. C., Van Timmeren, S., & Bruck, D. J. (2015). Infestation of wild and ornamental non- crop fruits by *Drosophila suzukii* Matsumura (Diptera: Drosophilidae). Ann Entomol Soc Am. 2015;108: 117-129.

34. Evans RK, Toews MD, Sial AA. Diel periodicity of *Drosophila suzukii* (Diptera: Drosophilidae) under field conditions. PLoS ONE. 2017;12: e0171718.

35. Leach H, Grieshop MJ, Isaacs R. Integrated strategies for management of spotted wing drosophila in organic small fruit production. 2016 Dec 13. Available from: https://www.canr.msu.edu/resources/integrated_strategies_for_management_of_spotted_wing_drosophila_in_organic

36. Kamiyama MT, Guédot C. Varietal and developmental susceptibility of tart cherry (Rosales: Rosaceae) to *Drosophila suzukii* (Diptera: Drosophilidae). J Econ Entomol. 2019;112: 1789-1797.

Supporting information

S1 Fig. Weekly adult *D. suzukii* trap catch separated by site over degree days. Relationship between log-normalized adult *D. suzukii* total trap catch for each orchard site/week and weekly cumulative degree days over the four year trapping period. A smoothed fit line shows approximate phenology of *D. suzukii* for each orchard in this dataset.

Figure 1

Click here to access/download;Figure;Fig1.tiff

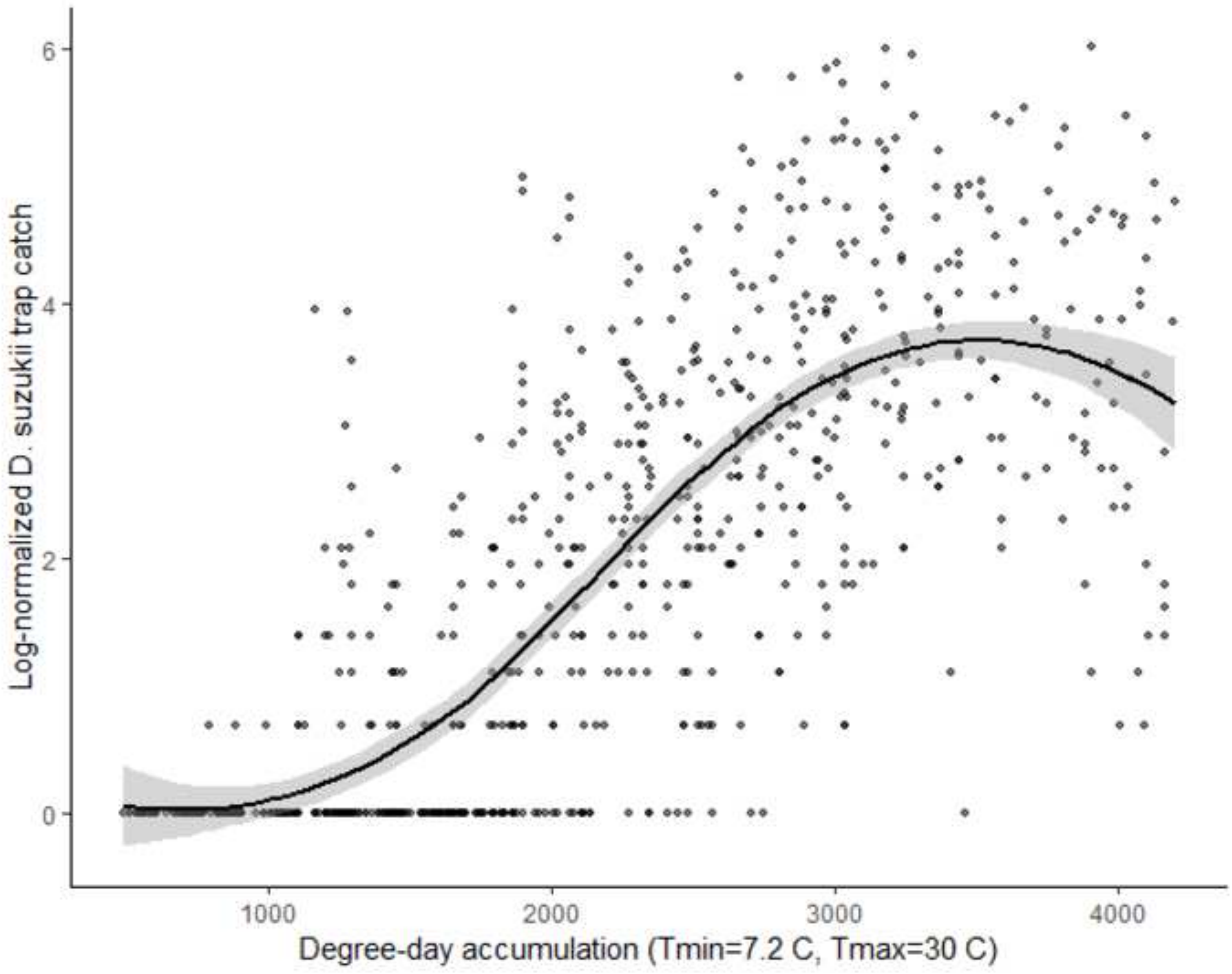

Figure 2

Click here to access/download;Figure;Fig2.tiff

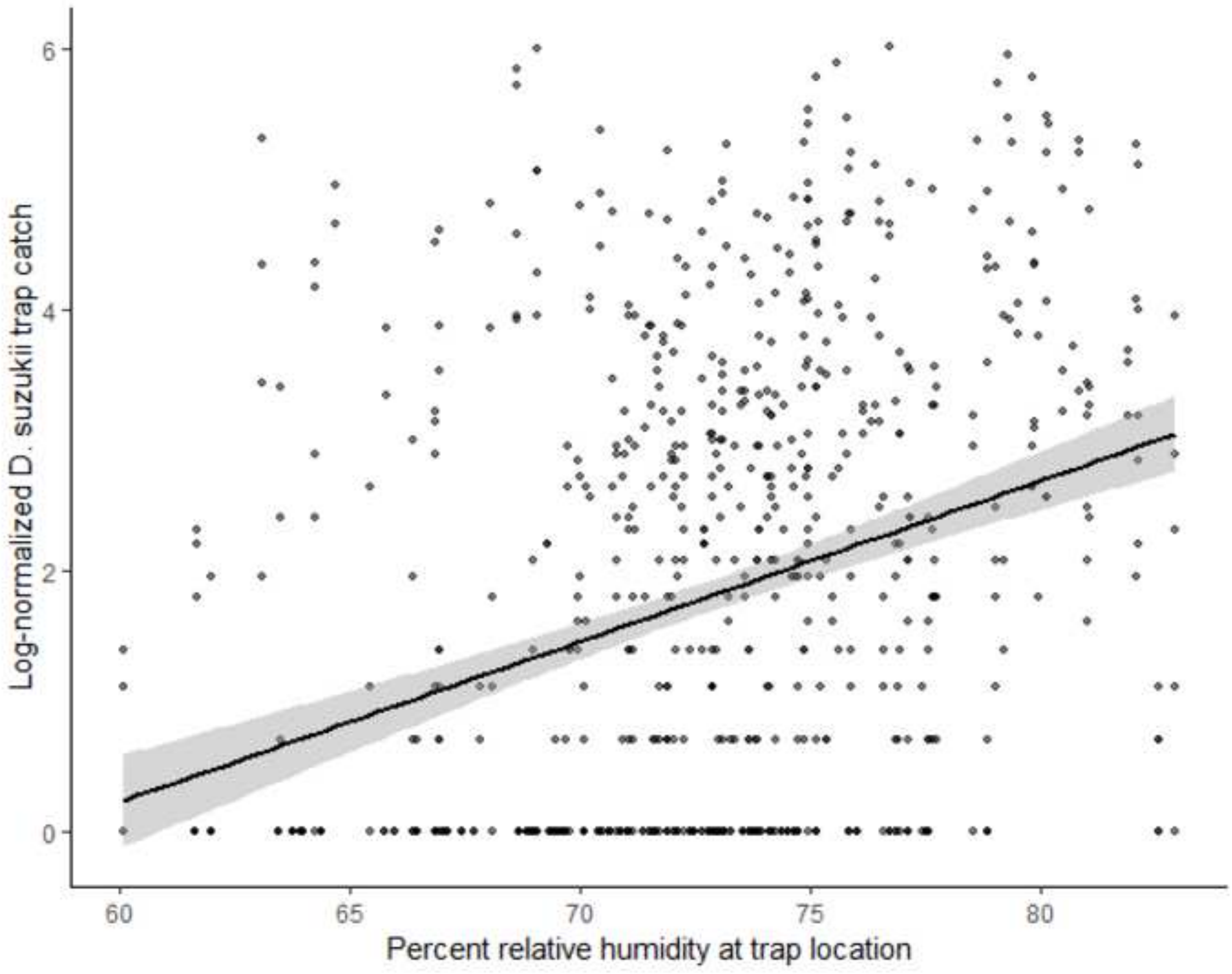

Figure 3

Click here to access/download;Figure;Fig3.tiff

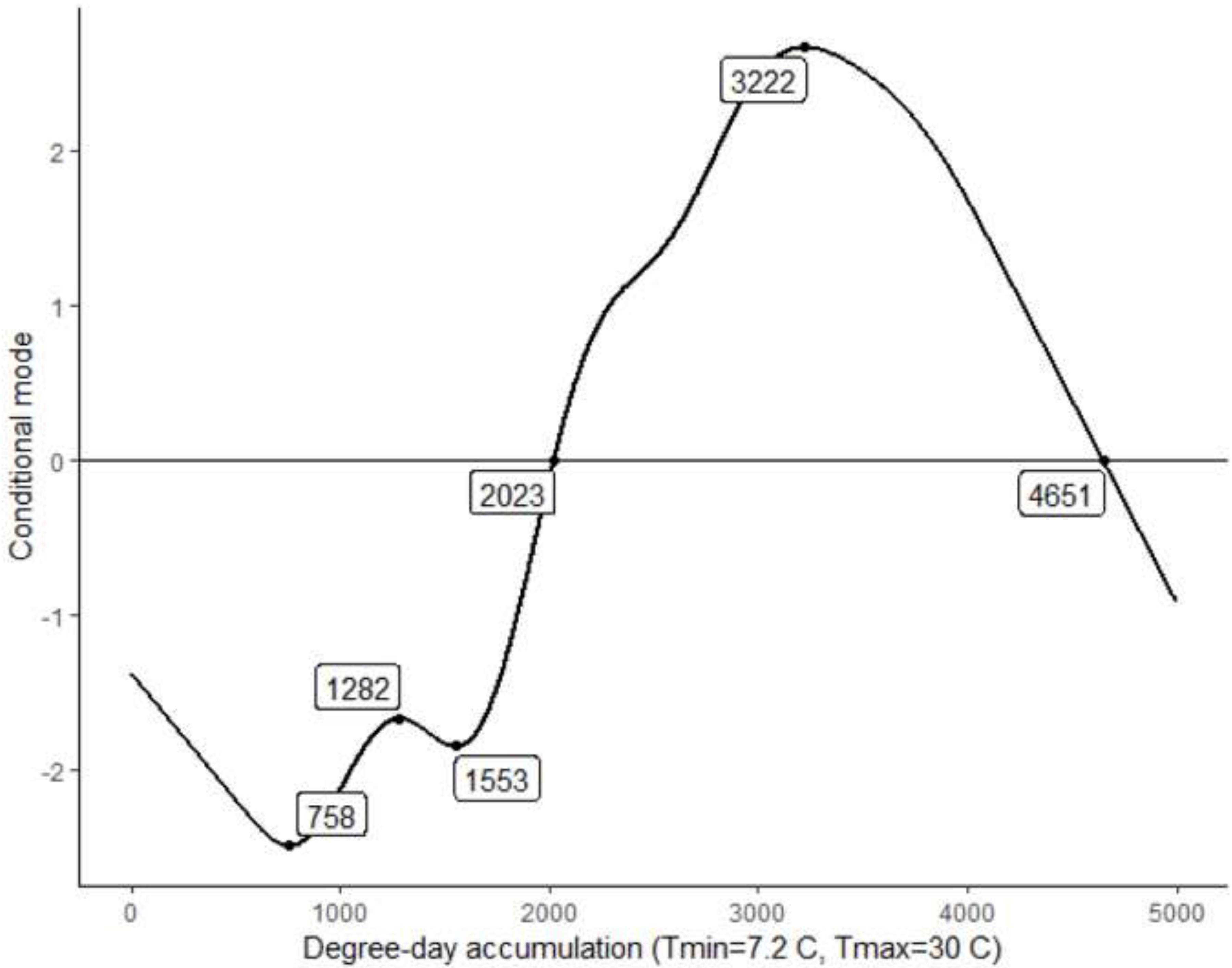

Supporting Information Figure

Click here to access/download
Supporting Information
SFig1.tiff

Data

Click here to access/download
Supporting Information
Raw Data.xlsx
