# Supplementary material for: Degree day models to forecast the seasonal phenology of Drosophila suzukii in tart cherry orchards in the Midwest U.S
Source: PLoS One. 2020 Apr 24;15(4):e0227726. doi: 10.1371/journal.pone.0227726 (PMC7182266; doi:10.1371/journal.pone.0227726)
Supplement: Attachment — Submitted filename: PONE-D-19-35638_notes.pdf [file pone.0227726.s007.pdf]

• Highlight, page 9
L28 prominent - common?

• Highlight, page 9
L31 with five or more applications under heavy pressure

• Highlight, page 9
L35 risk of developing resistance

• Highlight, page 10
L46 heat units

• Highlight, page 10
L50 to coincide with predictions of

• Strike Out, page 10
L53 the

• Highlight, page 10
L54 any reasons why phenology might be different in the midwestern US?

• Highlight, page 11
L69 would be nice to report GPS points for sites, latitude info could prove useful to others

• Highlight, page 11
L88 What resolution and time scale were the PRISM data?

• Highlight, page 11
L90 degree-days (DD) - what method used for DD?

• Highlight, page 13
L33 but wouldn't this be more in the SWD foraging environement rather than ambient? Were the orchards irrigated?

• Strike Out, page 14
L154 we?

• Highlight, page 16
L183 explanation for the confidence interval?

• Highlight, page 16
L186 explanation for CI missing

• Highlight, page 17
L206 smoothing protocol? Loess?

• Highlight, page 18
L244 but presumably if there was a later crop or host, the populations would likely increase well beyong the monitored period (Oct), or at least you don;t know from this data set, these data are specific to the tart cherry crop and should not be expanded to generalize the whole population

• Highlight, page 21
L297 Careful, no data on crop infestation are presented. What if damage preceded trap captures? Unhappy growers.

• Highlight, page 21
L309 Would be important to link the model to 1) crop susceptibility (color), 2) infestation/damage.
